# Prevention of radiation-induced liver toxicity after interstitial HDR brachytherapy by pentoxifylline and ursodeoxycholic acid: patient compliance and outcome in a randomized trial

**DOI:** 10.1007/s00432-023-04832-w

**Published:** 2023-05-11

**Authors:** Robert Damm, Joanna Wybranska, Peter Hass, Mathias Walke, Jazan Omari, Maciej Pech, Ricarda Seidensticker, Jens Ricke, Max Seidensticker

**Affiliations:** 1 Department of Radiology and Nuclear Medicine, Otto-Von-Guericke-University Magdeburg, Magdeburg, Germany; 2 Department of Radiation Oncology, Helios Klinikum, Erfurt, Germany; 3 Department of Radiation Oncology, Otto-Von-Guericke-University, Magdeburg, Germany; 4 Department of Radiology, Ludwig-Maximilians-University Munich, Munich, Germany

**Keywords:** Radiation-induced liver disease, Interstitial brachytherapy, Liver toxicity, Local ablative treatment, Liver cancer

## Abstract

**Aim:**

To investigate the impact of pentoxifylline (PTX, 3 × 400 mg per day) and ursodeoxycholic acid (UDCA, 3 × 250 mg per day) administered for 12 weeks on radiation-induced liver toxicity.

**Materials and methods:**

Inclusion criteria were liver metastases of extrahepatic malignancies undergoing HDR-BT. 36 patients were prospectively randomized to the

medication (*N* = 18) or control arm (*N* = 18) and follow-up by hepatobiliary magnetic resonance imaging (MRI) was scheduled 6 and 12 weeks after local ablation by HDR-BT. We determined the threshold doses of fRILI by image fusion of MRI with the dosimetry data.

**Results:**

32 patients completed the study schedule. Per-protocol treatment was limited to 8 patients in the medication group and 16 patients in the control group. 22 adverse events of any grade likely or certainly related to PTX were recorded in 12 patients leading to the discontinuation of the study medication in 7 patients and to a dose reduction of PTX in 2 patients. In the per-protocol population, statistical analysis failed to prove a reduction of fRILI 6 and 12 weeks after HDR-BT. The incidence of adverse effects attributed to PTX (70.6%) was well above the data found in the literature for its approved indication.

**Conclusion:**

The study endpoint was not met mainly attributed to the low statistical power of the small per-protocol cohort. Independently, PTX cannot be recommended for the reduction of radiation-induced liver toxicity in oncologic patients undergoing HDR-BT of liver metastases. Further studies might focus on a combination of UDCA with other potential drugs to help establish a preventive and tolerable regimen.

## Introduction

Various forms of radiotherapy are available for the treatment of liver malignancies today. Stereotactic body-radiotherapy (SBRT), Y90-radioembolisation (Y90-RE) and interstitial brachytherapy (iBT) are known to be effective in liver oligometastases as well as primary liver tumors (hepatocellular carcinoma and cholangiocarcinoma) (Baere et al. 2017; Jung et al. 2019; Magistri et al. 2017).

Radiation or radioembolization induced liver disease (RILD or REILD) is a limiting factor of these treatment modalities and originates from the sinusoidal obstruction syndrome, a condition also known to occur after certain chemotherapies or bone marrow transplantation (Lawrence et al. 1995; Sangro et al. 2008). It is characterized by a congestion of hepatic sinusoids and central veins by deposits of extracellular matrix leading to a hepatocyte necrosis and was formerly known as the veno-occlusive disease (VOD) (DeLeve et al. 2002; Fan and Crawford 2014). Clinical presentation of SOS/VOD or RILD/REILD includes jaundice, weight gain and hyperbilirubinemia in affected patients (Kumar et al. 2003; Mohty et al. 2016). In high-conformal radiotherapy (i.e., SBRT or iBT), focal radiation-induced liver injury (fRILI) can be visualized by hepatobiliary magnetic resonance imaging (MRI) and quantified in relation to a specific isodose by image fusion with the 3D irradiation treatment plan (Ricke et al. 2005; Seidensticker et al. 2011). In other terms, this setting allows to identify the isodose reflecting radiation tolerance of liver tissue.

In a prior randomized trial, the effect of pentoxifylline (PTX), ursodeoxycholic acid (UDCA) and low molecular weight heparin (LMWH) as a potential treatment of RILD/REILD by the occurrence and extent of fRILI after interstitial brachytherapy (iBT) of liver metastases was analyzed (Seidensticker et al. 2014). Study medication was administered for 8 weeks (treatment group) and hepatobiliary MRI with subsequent determination of isodoses revealed a significant reduction of fRILI 6 weeks after radiation compared to the control group (19.06 ± 3.35 Gy vs. 14.64 ± 4.01 Gy; *p* = 0.011). As a combination regimen was tested, it remained unclear which study drug had the biggest impact on fRILI. Furthermore, a prolonged liver injury was seen after 3 months in the treatment group suggesting the administration period of 8 weeks being too short while the cellular mechanisms of SOS/VOD seemed to continue.

In the recent trial, we altered the study protocol by removing LMWH and prolonging the administration of PTX and UDCA to 3 months to gain further information on the development of fRILI.

## Materials and methods

### Study design and inclusion criteria

In this prospective trial sequel, we employed a parallel-group design with 1:1 randomization to the treatment and control group. All patients gave written informed consent prior to randomization.

Key inclusion criteria were: (i) patients with liver metastases in non-cirrhotic liver scheduled for interstitial brachytherapy; (ii) age 18–85 years and performance status ECOG 0 or 1; (iii) no prior irradiation therapy of the liver; (iv) no restricted renal function (estimated glomerular filtration rate < 45 ml/min) and (v) no oral anticoagulants.

### Patient cohort and study medication

**Fig. 1:**
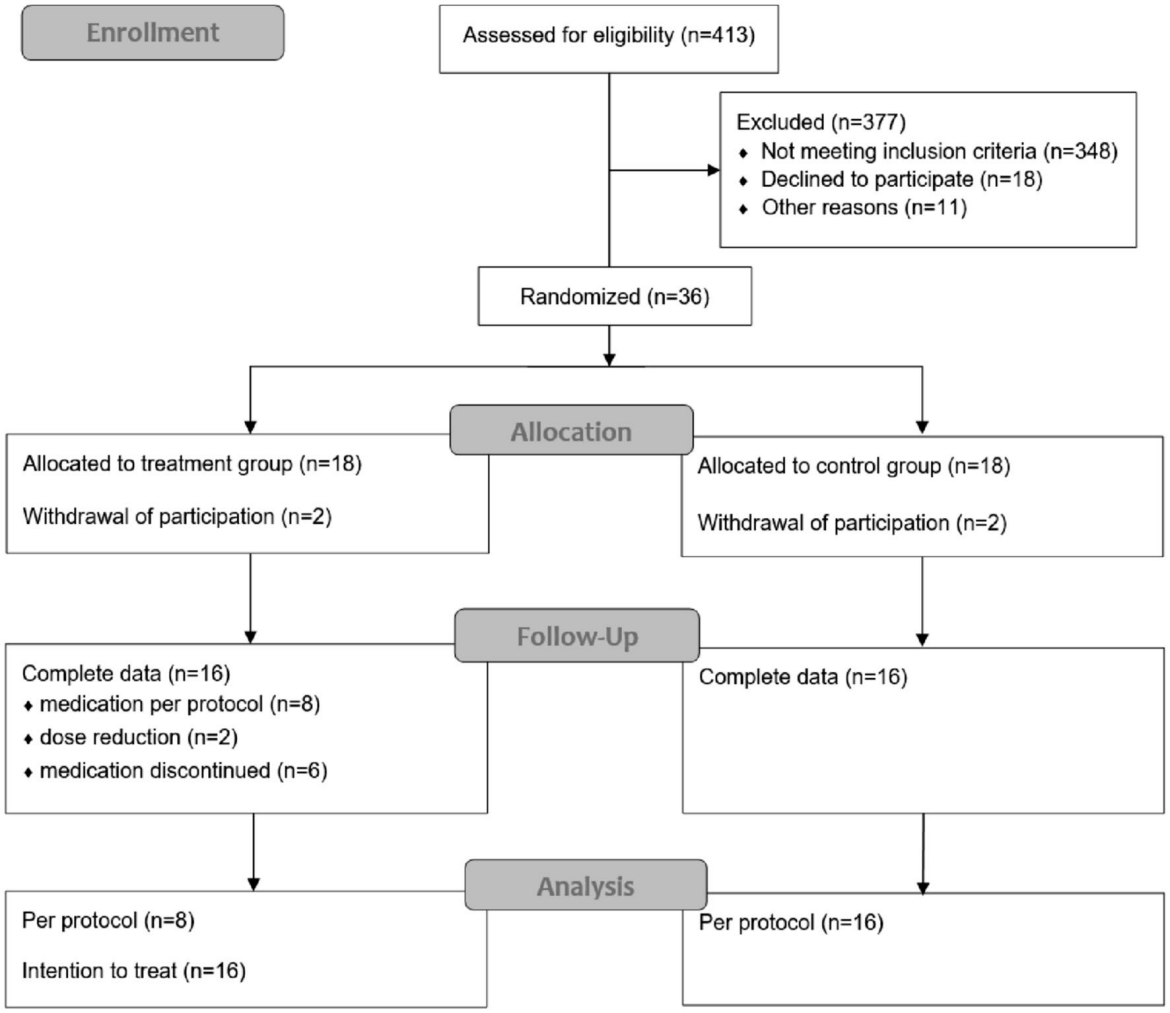
CONSORT diagram of the study

An overview of the study population in given in the CONSORT diagram (see Fig. 1).

We enrolled a total of 36 patients (18 males, 18 females, mean age 64.0 years) in our Department of Radiology and Nuclear Medicine with 18 being assigned to each study group after randomization. In the treatment arm, patients were scheduled to receive the medication beginning the first day after interstitial brachytherapy for 3 months: PTX (Trental, Sanofi Aventis, Frankfurt, Germany) 400 mg t.i.d. oral and UDCA (Ursofalk, Falk Pharma, Freiburg, Germany) 250 mg t.i.d. oral. As before, patients and physicians were not blinded for the study medication. All drugs were supplied by the local pharmacy. Treatment compliance was monitored by an interrogation at each visit while personal contact to an investigator and/or study nurse was possible any day during regular working hours.

### Treatment by interstitial brachytherapy

All interventions were performed under conscious sedation using Fentanyl and Midazolam: initial puncture of the target volume was achieved under CT or MRI fluoroscopy guidance with an 18G coaxial needle. Subsequently, 6F catheter sheaths (Terumo Radifocus^®^ Introducer II, Terumo Europe, Leuven, Belgium) and 6F irradiation catheters (afterloading catheter, Primed^®^ medical GmbH, Halberstadt, Germany) were inserted in Seldinger’s technique through a stiff guidewire (Amplatz SuperStiff™, Boston Scientific, Marlborough, USA). Multiple catheter placements were typically required in larger (> 3 cm) or complex lesions to account for a sufficient dose distribution while lowering exposure to organs at risk.

After positioning of the catheters, a 3D treatment plan was created based on a contrast-enhanced CT or MRI scan with 3 mm slice thickness in an irradiation planning system (Oncentra^®^ Brachy, Elekta Instrument AB, Stockholm, Sweden). Depending on the cancer entity, dose prescription for the planning target volume (PTV) was a single fraction of 25 Gy for colorectal cancer metastases, 20 Gy for cholangiocarcinoma and 15 Gy for metastases of other origin (e.g., breast cancer) applied by afterloading (Jonczyk et al. 2018; Mohnike et al. 2010; Ricke et al. 2010; Wieners et al. 2011).

Catheters and sheaths were then removed with application of gelatin sponge to the catheter path to prevent postinterventional bleeding.

### Data acquisition and follow-up

Patient data including imaging (i.e., MRI), liver specific and inflammatory laboratory parameters as well as quality of life (according to the EQ-5D questionnaire) were collected at baseline as well as 6 weeks, 3 and 6 months after iBT.

Patients interrupting the study medication for 7 or more days were excluded from the primary analysis. Furthermore, occurrence of adverse events ≥ °II according to the Common Terminology Criteria for Adverse Events (CTCAE 4.02) led to a discontinuation of the study drugs for safety purposes.

Processing of the imaging data was performed as in the prequel study (Seidensticker et al. 2014). In short, visualization of fRILI was achieved in a 1.5 T MRI scanner (Achieva, Philips, Best, The Netherlands) using the hepatocyte-specific contrast agent Gd-EOB-DTPA (Primovist, Bayer Healthcare, Leverkusen, Germany) and axial 3D T1-weighted gradient echo sequences (T1 high resolution isotropic volume excitation) 20 min after contrast administration. Then, image fusion was performed by semi-automated point-based image registration with the irradiation plan. Isodoses corresponding to the demarcation of fRILI were recorded by two methods (dose-volume-histogram and five axial slices) using the mean for further analysis. The threshold dose of fRILI 6 weeks after iBT was determined the primary efficacy variable.

All measurements within this process were blinded for the study group.

### Statistical analysis

We employed a Welch design without interim analyses based on the data acquired in the prequel study assuming a 4.5 Gy difference in threshold isodoses of fRILI between patient groups (Seidensticker et al. 2014), number of patients was calculated accordingly with a one-sided statistical significance of *p* < 0.025 and a power of 80%. Patients with insufficient compliance in the treatment cohort were not replaced.

Permuted block randomization was performed at the local Institute of Biometry using the Mersenne twister in RITA 1.24, Evidat^®^ Statistical Apps. Determined isodoses reflecting fRILI (primary efficacy variable) were compared using a one-sided *t* test with significance assumed for *p* < 0.025. Analysis of further variables was carried out by a *t* test for parametric variables and fishers exact test for frequencies, respectively. Quality of life was compared by the Mann–Whitney test. As no prior calculation was available, these tests were carried out two-sided and significance was assumed for *p* < 0.05.

All statistical analyses were performed in IBM SPSS Statistics 22.0^®^.

## Results

### Study population and patient characteristics

Out of 413 patients scheduled for interstitial brachytherapy of liver malignancies (e.g., colorectal liver metastases), 36 patients met the inclusion criteria and volunteered in participation. Two patients withdraw participation in each group shortly after informed consent leading to 16 patients entering the regular study schedule in the treatment and control arm, respectively. Required data acquisition were achieved in 16 patients per group.

All patients had up to three liver metastases of various malignancies (colorectal carcinoma *N* = 24; breast cancer *N* = 4; gastric cancer *N* = 3; neuroendocrine tumor, cholangiocarcinoma, lymphangiosarcoma *N* = 1 each). Distribution of genders was equal with 8 males and 8 females in each study group, patient’s age ranged from 47 to 84 years (mean 64.0 years) with no significant difference between groups. Total liver volume and sum of clinical target volumes were well balanced. A summary of the study population is given in Table 1.

**Table 1:** Intention to treat: patient characteristics of the study population including treatment characteristics and adverse events (AE)

Variable	Treatment arm (*N* = 16)	Control arm (*N* = 16)	*p* value*
Sex (m/f)	*N* = 8/*N* = 8	*N* = 8/*N* = 8	1.0
Age (years)	63.6 ± 9.9	64.5 ± 13.0	0.83
Colorectal liver metastases (y/n)	*N* = 12/*N* = 4	*N* = 12/*N* = 4	1.0
No. of metastases	1.25 ± 0.56	1.25 ± 0.46	1.0
Total liver volume (ml) at baseline	1241.3 ± 289.7	1444.6 ± 413.0	0.12
Total clinical target volume (ml)	25.2 ± 36.3	33.2 ± 45.5	0.61
fRILI volume (ml)			
6 weeks	63.2 ± 77.7	82.3 ± 95.6	0.58
3 months	53.5 ± 67.2	68.5 ± 68.3	0.58
No. of AE (all grades)	18	4	**0.021**
Max. severity of AE			
°I	*N* = 4	*N* = 3	
°II	*N* = 6	*N* = 1	
°III	*N* = 1	–	**0.04**

*p < 0.05 was defined as statistically significant

### Adverse events and treatment compliance

17 patients initiated treatment with the study medication, yet one patient canceled participation within 24 h to personal reasons. Per-protocol treatment (3 months of PTX and UDCA as prescribed, not more than 7 days of interruption) was only accomplished in 8 patients (47.1%), while a dose reduction had to be accepted in 2 individuals (11.8%). As 12 patients (70.6%) experienced adverse events attributed to PTX administration, discontinuation of treatment with PTX was necessary in 7 patients (41.2%).

In the study cohort, a total of 22 adverse events of any grade were recorded within the observation period, typically within one week after initiation of the study-specific medication: abdominal pain *N* = 6, nausea *N* = 5, vomiting *N* = 3, headaches and hemorrhages each *N* = 2, tachycardia, diarrhea, hand-foot-syndrome and dysgeusia each *N* = 1. A majority of events (*N* = 18) was likely or certainly attributed to PTX as not caused by the conduct of interstitial brachytherapy within a reasonable time after intervention (*N* = 4). Furthermore, all these findings resolved after discontinuation of PTX.

Severity of events according to CTCAE (patients of treatment arm vs. control arm) was grade 1 in 7 patients (*N* = 4 vs. *N* = 3), grade 2 in 7 patients (*N* = 6 vs *N* = 1) and grade 3 in one patient of the treatment arm. Comparing both groups with Fishers exact test, events in the treatment arm were significantly more frequent (*p* = 0.021) and severe (*p* = 0.04).

Adverse events in the investigative arm and expected frequency of side effects according to the summary of product characteristics (SmPC) are depicted in Table 2. As the overall incidence was obviously increased in our study population, a safety report was submitted to the competent authority and the trial stopped.

**Table 2:** Adverse events of any grade in the investigative arm attributed to PTX (in 17 patients exposed to PTX and UDCA) and expected frequency as stated in the summary of product characteristics (SmPC) of PTX as approved by the competent authority

Adverse event attributed to PTX	No. of events in 17 exposed patients	Frequency in 17 exposed patients	According to SmPC
Headache	*N* = 2	**11.8%**^a^	0.1–1%
Vomiting	*N* = 3	**17.6%**^a^	1–10%
Nausea	*N* = 5	**29.4%**^a^	1–10%
Abdominal pain	*N* = 6	**35.3%**^a^	1–10%
Tachycardia	*N* = 1	**5.8%**^a^	0.1–1%
Dysgeusia	*N* = 1	**5.8%**^a^	Not mentioned
Diarrhea	*N* = 1	5.8%	1–10%
Hand-foot-syndrome	*N* = 1	**5.8%**^a^	0.1–1%
Hemorrhaging	*N* = 2	**11.8%**^a^	0.001–0.1%

^a^Frequency in the investigative arm above expected frequency according to the SmPC

### Primary efficacy of PTX and UDCA

The primary efficacy variable was the isodose of fRILI as depicted by hepatobiliary MRI 6 weeks after iBT. In the per-protocol population, only 24 patients out of 36 (66.7%) could be included for reasons mentioned above—the determined isodose was not statistically different between the groups with 13.8 ± 5.6 Gy (investigative group, *N* = 8) vs. 14.5 ± 5.6 Gy (control group, *N* = 16) utilizing Student’s *t* test (*p* = 0.76). Same was seen for the intention-to-treat analysis with 14.5 ± 5.4 Gy (investigative group, *N* = 116) vs. 14.5 Gy ± 5.6 Gy (control group, *N* = 16) and *p* = 0.98.

Restricting the analysis to the subgroup of patients with metastasized colorectal carcinoma similar to our prequel study, neither was a significant difference found (14.8 ± 6.5 vs. 15.0 ± 4.1 Gy, *p* = 0.96). Furthermore, no difference in fRILI could be seen 3 and 6 months after iBT between both groups, see Table 3.

**Table 3:** Per protocol: treshold doses of fRILI determined by image fusion of hepatobiliary MRI and 3D irradiation planning and liver function according to serum bilirubin (means with standard deviation) compared between study groups

Variable	Treatment arm (*N* = 8)	Control arm (*N* = 16)	*p* value*
fRILI treshold isodose [Gy]			
6 weeks^a^	13.8 ± 5.6	14.5 ± 5.6	0.76
3 months	17.8 ± 4.2	16.8 ± 5.8	0.69
6 months	21.0 ± 4.7	20.2 ± 5.5	0.83
Serum bilirubin [µmol/l]			
Baseline	7.6 ± 3.2	8.7 ± 4.1	0.41
6 weeks	6.9 ± 2.4	8.5 ± 4.1	0.22
3 months	8.6 ± 6.5	7.5 ± 3.2	0.61

*p < 0.025 in one-sided analyses and *p* < 0.05 in two-sided analyses were defined as statistically significant

^a^Primary efficacy variable

### Laboratory evaluation and quality of life

As a surrogate marker of liver function, we analysed bilirubin serum levels at baseline as well as 6 weeks and 3 months after iBT as means with standard deviation by Student’s *t* test.

At all points in time, means were within normal ranges (< 21 µmol/l), comparison between groups and within each group did not show any statistical significance, see Table 3.

Similar analyses were carried out for other liver specific parameters (albumin, alanine-amino-transferase, aspartate-amino-transferase, glutamate-dehydrogenase and choline-esterase) and did not reveal any significant elevation compared to baseline as well.

Quality of life (QoL) was assessed according to the EQ-5D questionnaire as percentages. Mean overall QoL was 69.2 ± 16.6% at baseline, 70.0 ± 18.1% after 6 weeks and 72.9 ± 13.7% after 3 months. Analysis by independent samples Mann–Whitney test to compare both groups demonstrated p-values between 0.52 and 0.72. Thus, no significant influence of the study group on QoL was seen.

## Discussion

As the radiation tolerance of healthy liver parenchyma is a limiting factor of ablative radiation therapies (e.g., interstitial brachytherapy or stereotactic body radiotherapy) targeting liver malignancies, a medication-based increment of hepatic tolerance doses might play a substantial role to increase dose delivery to the target volume.

In a prior study, we investigated ursodeoxycholic acid (UDCA), pentoxifylline (PTX) and low molecular weight heparin (LMWH) administered for 8 weeks in patients undergoing interstitial brachytheraphy (iBT) of colorectal liver metastases (Seidensticker et al. 2014). Analysis of functional radiation-induced liver injury (fRILI) as detected by hepatobiliary MRI and correlated to the 3D irradiation plan revealed a significant increase of mean hepatic tolerance doses (19.1 vs. 14.6 Gy, *p* = 0.011) 6 weeks after irradiation. Driven by the positive study results of this prequel study, we continued the investigation by omitting the medication with LMWH and extending the medication period with UDCA and PTX to 3 months after iBT.

Here, we encountered an unexpected accumulation of adverse events arising typically within one week after initiation of medication attributed to PTX (e.g., nausea up to 30% and abdominal pain up to 35% of patients) which resolved within two days after discontinuation and did not require additional medication. As side effects of UDCA only include alteration of stool quality and no interactions with PTX are assumable according to the pharmacological properties, a cumulative effect of both drugs is unlikely.

Although the number of patients in the recent study is low, a total of eight different conditions (all known to be side effects of PTX) were recorded and frequencies were at least two time above the expected frequency (according to SmPC) in six of them (SmPC Trental 2023). As the study medication had a prophylactic intent, even mild or moderate toxicities (95% grade 1/2) lead to an early discontinuation at the patient’s discretion. This also explains that no difference in quality of life was recognized in the 6 weeks or 3 months follow-up.

In total, susceptibility to side effects of PTX may be increased in patients with metastases of solid tumors which should be regarded as a cohort at risk compared to patients with peripheral artery disease (regular indication of PTX). Possible explanations of these findings might include prior history of chemotherapy or concomitant interstitial brachytherapy while no similar reports can be found in the literature on bone marrow transplantation (Beelen et al. 1993; Bianco et al. 1991; Jagt et al. 1994).

As a consequence, only 8 out of 16 patients in the investigative arm were included in the per-protocol analysis which could not demonstrate the expected effect of PTX and UDCA on radiation-induced liver dysfunction. Beside the small number of eligible patients resulting in a low statistical power, reduction of the combination treatment by LMWH must be assumed to have contributed to the negative outcome of our study.

Investigational drugs potentially preventing fRILI still originate from studies of veno-occlusive disease in the setting of bone marrow transplantation (included whole-body irradiation in the conditioning regimen) (Attal et al. 1993; Ohashi et al. 2000; Or et al. 1996), while reports on recent radiotherapies of liver malignancies (e.g., stereotactic body radiotherapy, selective internal radiation therapy) lack a systematic evaluation of pharmacological regimen utilized to prevent fRILI (Koay et al. 2018). Furthermore, low-volume exposition of healthy liver parenchyma found in high-conformal irradiation techniques requires a surrogate (e.g., hepatobiliary MRI) as the marginal deterioration of global liver function stays subclinical in most patients. Accordingly, no single agent can be recommended as a standard of care in modern radiation-based therapies of liver malignancies (Sangro et al. 2017; Toesca et al. 2018). Taking all available data into account, we hypothesize best results for a combination of LMWH and UDCA possibly complemented by corticosteroids (e.g., Cortisone) in future investigations (Mohty et al. 2020).

## Conclusion

Based on our small study cohort, we cannot rule out that PTX might provoke adverse events more frequent in oncologic patients compared to its original approval in peripheral artery disease.

Yet, efficacy of PTX in combination with UDCA in the prevention of radiation-induced liver toxicity after interstitial brachytherapy of liver malignancies could not be evaluated thoroughly due to poor patient compliance caused by substantial side effects. As a preventive effect on liver dysfunction after irradiation was found in a prior cohort receiving LMWH, PTX and UDCA, further studies should focus on a combination treatment with of LMWH and UDCA.

## Data Availability

The datasets generated and analysed during the current study are available from the corresponding author on reasonable request.
